# High-resolution SNP genotyping provides insight into the introduction and dissemination of Grapevine flavescence dorée phytoplasma in Switzerland

**DOI:** 10.3389/fpls.2026.1761458

**Published:** 2026-02-06

**Authors:** Jasmine Cadena i Canals, Christophe Debonneville, Nathalie Dubuis, Isabelle Kellenberger, Léa Mandelli, Michel Jeanrenaud, Olivier Viret, Olivier Schumpp

**Affiliations:** 1 Virology, Bacteriology and Phytoplasmology, Agroscope, Nyon, Switzerland; 2 Department of Agriculture, Viticulture and Veterinary Affairs (DGAV) Canton of Vaud, Morges, Switzerland

**Keywords:** flavescence dorée phytoplasma, genetic diversity, MLST, molecular epidemiology, SNP genotyping

## Abstract

Flavescence dorée is a quarantine grapevine disease transmitted by Scaphoideus titanus that was first detected north of the Swiss Alps in 2015 and has since progressively spread in western Switzerland, while its introduction routes and local dissemination dynamics remained poorly understood. To clarify these processes, we applied a molecular epidemiology approach combining MLST based on established markers (*map, dnaK, vmpA, malG*) with high-resolution SNP genotyping using newly developed loci to investigate FDp spatiotemporal dynamics and genetic diversity. Between 2015 and 2022, 4,212 symptomatic grapevines were sampled across Vaud, Valais, and Geneva, of which 26.3% tested positive for FDp. Established markers revealed highly homogeneous profiles, with all samples sharing the same genotypes (map M54, dnaK1, vmpA group II), supporting introduction through infected planting material. The *malG* locus alone distinguished only three profiles, but when combined with eight novel SNP markers, nine multilocus genotypes were identified, providing a finer resolution of FDp population structure. Our results provide the first genome-informed overview of FDp epidemiology in Swiss vineyards north of the Alps, highlighting the combined roles of planting material and local dissemination in shaping epidemic dynamics. The newly identified SNP markers enhance genotyping resolution and provide valuable tools for investigating phytoplasma spread and improving future surveillance strategies.

## 1 Introduction

Identifying the source of an epidemic outbreak and tracing its spread is a challenging task, yet essential for understanding the nature and drivers of the epidemic and ultimately for containing it. This is specifically relevant for quarantine diseases such as flavescence dorée (FD). This disease affects grapevines and is associated with Grapevine flavescence dorée phytoplasma (FDp), provisionally named *Candidatus* Phytoplasma vitis, which is naturally transmitted by the leafhopper *Scaphoideus titanus* Ball (Schvester et al., 1961). The presence of this vector enables epidemic outbreaks. FD causes substantial yield and economic losses (Oliveira et al., 2020; Ripamonti et al., 2022). As there is no curative treatment, and because FDp is a regulated quarantine pathogen (European Union, 2019), control strategies are legally mandated. These include insecticide treatments against *S. titanus*, imposing substantial environmental and financial burdens, as well as annual surveys and the compulsory uprooting of infected plants, resulting in significant agronomic and economic consequences. Improving our understanding of the disease’s epidemiology and dissemination pathways is therefore essential for the development of more targeted, effective, and environmentally responsible containment strategies.

FDp is endemic to different European plant species that act as alternative natural reservoirs (Malembic-Maher et al., 2020). It is thought to have spread from alders (*Alnus glutinosa*) and climbing shrubs (*Clematis vitalba*) to grapevine via leafhoppers that occasionally fed on *Vitis* (Arnaud et al., 2007; Filippin et al., 2009). Additional plant species such as *Corylus avellana* (Casati et al., 2017; Kogej Zwitter et al., 2023) and *Ailanthus altissima* (Filippin et al., 2011; Rigamonti et al., 2023) have also been found to host FDp genotypes implicated in vineyard outbreaks, and other studies have reported its detection in green manure crops and other wild plants (Moussa et al., 2023; Rigamonti et al., 2023), although their role as epidemiologically relevant hosts remains uncertain. Beyond these natural pathways, FDp can also be introduced through infected planting material. This route is particularly problematic because rootstocks, while susceptible, remain asymptomatic (Eveillard, 2016), allowing FDp to spread silently over long distances. Together, these findings indicate that FDp can enter vineyards either from surrounding reservoirs or via infected propagation material. High-resolution genotyping of FDp strains is therefore essential to link genotypes to hosts and reconstruct epidemiological connections across localised outbreaks.

Recent advances in the identification of hypervariable regions within the FDp genome have significantly improved our ability to explore the diversity of genotypes involved in grapevine outbreaks. Among this, the *map* locus shows particularly high variability, with more than 170 genotypes identified, and allows classification into three groups: Map-FD1, Map-FD2 and Map-FD3 (Arnaud et al., 2007; Malembic-Maher et al., 2020). This marker is particularly relevant for epidemiological tracking, as it distinguishes genotypes found exclusively in the grapevine - *S. titanus* pathosystem (namely M12 and M54) from those also present in other plant-vector associations such as *Alnus glutinosa - Oncopsis alni* or *Clematis vitalba - Dictyophara europea*. Another key marker, *vmpA*, encodes for a membrane protein that facilitates the phytoplasma adhesion to the insect midgut. This gene plays a critical role in determining vector compatibility and thus epidemic potential (Malembic-Maher et al., 2020). Only strains belonging to *vmpA* clusters II and III can complete the transmission cycle in grapevine, and are therefore classified as FDp. Finally, Rossi et al. (2019) further contributed to the characterization of FDp by identifying two additional loci: *dnaK*, which occurs in three types, and *malG*, a highly variable locus with up to 183 types. Notably, *malG* is duplicated in certain FDp strains, contributing to even greater genetic diversity.

Multilocus sequence typing studies (MLST) based on the above-mentioned markers have been carried out across the main infected zones in Europe, particularly in different Italian regions (Martini et al., 1999; Pierro et al., 2024, 2023; Rigamonti et al., 2023; Rossi et al., 2019), Serbia (Krstić et al., 2022), Slovenia (Kogej Zwitter et al., 2023), Montenegro (Radonjić et al., 2023) and Croatia (Plavec et al., 2019), as well as in European studies including France and other countries (Arnaud et al., 2007; Malembic-Maher et al., 2020). In Switzerland, some data have been published for the southern region of Ticino, where FDp has become established despite strict control measures, partly due to the presence of abandoned and gone-wild vineyards (Casati et al., 2017; Oggier et al., 2024, 2023). However, no comparable data are yet available for Swiss vineyards north of the Alps, where the disease was first reported in the canton of Vaud in 2015. Since then, several outbreaks have emerged, also in the cantons of Valais and Geneva. Despite strict control measures, the disease has continued to progress. It is therefore essential to better understand the mechanisms underlying FDp introduction and local dissemination in this region. Here, we aimed to elucidate the origin and spread of the ongoing FD epidemic in Swiss vineyards north of the Alps by combining spatial epidemiology with molecular genotyping. Given the limited discriminatory power of previously described markers in the Swiss context, we further applied a high-resolution SNP-based genotyping strategy.

## 2 Materials and methods

To enhance clarity and simplicity, we refer to SNP (single nucleotide polymorphism) in this work as any base variation relative to the reference genome CH - Genbank CP097583 (Debonneville et al., 2022), irrespective of its frequency within the population.

### 2.1 Plant material source

Over 4,200 grapevines showing yellows symptoms were geolocalised and sampled (fresh leaves) from 2015 to 2022 during official surveillance campaigns. Vegetal material was stored at 4 °C until total nucleic acids (TNA) extraction within three days of receipt.

### 2.2 Mapping epidemic dynamics and genotype clustering

Data were provided by the phytosanitary official services of the cantons. The geolocalisation of each plot containing positive samples was projected onto a map of the pertinent cantonal vineyard surfaces using the open-source software QGIS 3.34.2-Prizren. The data were organised and projected according to the year of detection to trace the progression of the epidemic.

The spatial distribution of *malG* genotypes was visualised using QGIS 3.34.2-Prizren. To improve map readability and prevent overlap between closely located samples, genotypes were aggregated within 3 × 3 km grid cells, and their relative frequencies were displayed as pie charts to summarise local diversity.

### 2.3 Total nucleic acids extraction and FDp detection

Approximately 1 g of leaf mid-vein tissue was used to extract total nucleic acids as previously described (Debonneville et al., 2022). FDp and Bois noir phytoplasma presences were tested by triplex quantitative PCR (qPCR) following a method adapted from (Pelletier et al., 2009).

### 2.4 Preparation of DNA samples for Illumina sequencing, data pre-processing, SNPs detection and analysis

TNA from seventeen plants were extracted and subsequently enriched with microbial DNA using NEBNext^®^ Microbiome DNA Enrichment Kit as described in (Debonneville et al., 2022). Illumina sequencing was performed by Macrogen (South Korea).

Raw Illumina reads were quality trimmed with the software Trimmomatic and mapped to FDp reference genome Bowtie 2 to exclude all reads not corresponding to FDp. Pre-processed Illumina reads were mapped to the reference genome with Bowtie2 and SNPs were detected using Geneious 2023.0.4. Parameters for variant calling were a minimum coverage of 10 and a minimum variant frequency of 80%. SNPs found in repeated regions or representing deletions and insertions in a base-repetition context were discarded due to low reliability.

### 2.5 MLST, primers and PCR amplification of target sequences

Genetic characterization was done by multilocus sequence typing. The number of samples successfully analysed varied among loci, ranging from 48 to 675, and is detailed for each locus in **Table 1**. Loci reported in the literature were amplified by PCR or nested PCR using published primers and amplification programs. Primers for newly identified SNPs were designed using Geneious 2023.0.4. All primers and references are listed in **Table 1**. PCR conditions for primers ftsH5, ftsH7, ftsH17, rpoC, pcrA, helicase, intergenic 38.423 and dnaX were 2 min at 94 °C, 30 s at 94 °C, 35 cycles with one cycle consisting of 30 s at 55 °C, 1 min 30 s at 72 °C and final extension of 10 min at 72 °C. All amplification reactions were performed using GoTaq ^®^ G2 Flexi polymerase (Promega), except for *malG* reactions, for which a Q5 High-Fidelity DNA Polymerase (New England Biolabs) was used.

**Table 1:** Primers used to amplify loci for multilocus sequence typing (MLST) of FDp.

Locus	Primers or reference	Number of samples genotyped
*map*	Arnaud et al., 2007	145
*dnaK*	Rossi et al., 2019	48
*vmpA*	Malembic-Maher et al., 2020	77
*malG*	Rossi et al., 2019	675
*ftsH5*	CCTGCTGAACCTCTTGGAAT (FW) GACCGTCAATTACATTTGAACCT (R)	119
*ftsH7*	CCTTTTCCACTTGTTGCACG (FW) TCCTGAATTATTCGCTACCGCT (R)	86
*ftsH17*	ACGAAAACAACTATTGAAGATCG (FW) TGTTGTTCTTCTTGAGTTTCAGC (R)	114
*dnaX*	CGGCGCATGATTCACAACAA (FW) AGTCTTCAAGCTGCTGGTTTT (R)	89
*pcrA*	AGAAGAATCGCTTATGTGGCGA (FW) TGTGCATAACCTTATCTCCTGCT (R)	86
*rpoC*	TGCAGGACGTTTTGCGACTA (FW) AAACGAGCTTCTGCTTGTGC (R)	89
*helicase*	TGCAAGCACTTTTTAAGCCG (FW) TGATAACGCTCAGGGTGACG (R)	77
Intergenic 38,423	AAACGGAATGTCTCAACAAGAATT (FW) TTTTTAAGTTTGACCATTTGGAAACA (R)	85

For each target locus, the corresponding primer sequences (forward and reverse), reference sources and the number of successfully analysed samples are provided.

### 2.6 Sanger sequencing and data analysis

After quality control by electrophoresis on 1% agarose gel, PCR products were purified by ultrafiltration with NucleoFast 96 PCR plates (Macherey-Nagel) and vacuum processing. Products were sent to Fasteris (Switzerland) for forward and reverse sequencing using Sanger technology. Raw sequences were trimmed and *de novo* assembled. Resulting consensus were multi-aligned using MUSCLE algorithm and compared to references.

### 2.7 Sequence translation

Expasy online tool^1^ was used for nucleotide sequence translation into protein to define whether SNPs produce synonymous or non-synonymous translations.

## 3 Results

### 3.1 Plant material and FDp detection

Between 2015 and 2022, a total of 4,212 grapevines exhibiting symptoms characteristic of grapevine yellows, including leaf discoloration and downward rolling, withering of inflorescences and berries, and lack of shoot maturation, were geolocated and sampled as part of the national surveillance program conducted by the phytosanitary services of the Cantons of Vaud, Valais, and Geneva (Switzerland). Samples were analysed by quantitative PCR (qPCR) for the detection of Grapevine flavescence dorée phytoplasma (FDp) and *Candidatus* Phytoplasma solani (Grapevine bois noir phytoplasma), two causal agents of grapevine yellows that produce identical symptoms and can only be differentiated by molecular assays. Of the tested samples, 26.26% were positive for FDp, 49.76% for Bois noir (BN) phytoplasma, 0.17% tested positive for both pathogens, and 23.81% were negative for both. These results highlight the predominance of BN among symptomatic grapevines in the surveyed regions between 2015 and 2022 and indicate that dual infections with both phytoplasmas are exceptionally rare.

### 3.2 Spatiotemporal distribution

**Figure 1:**
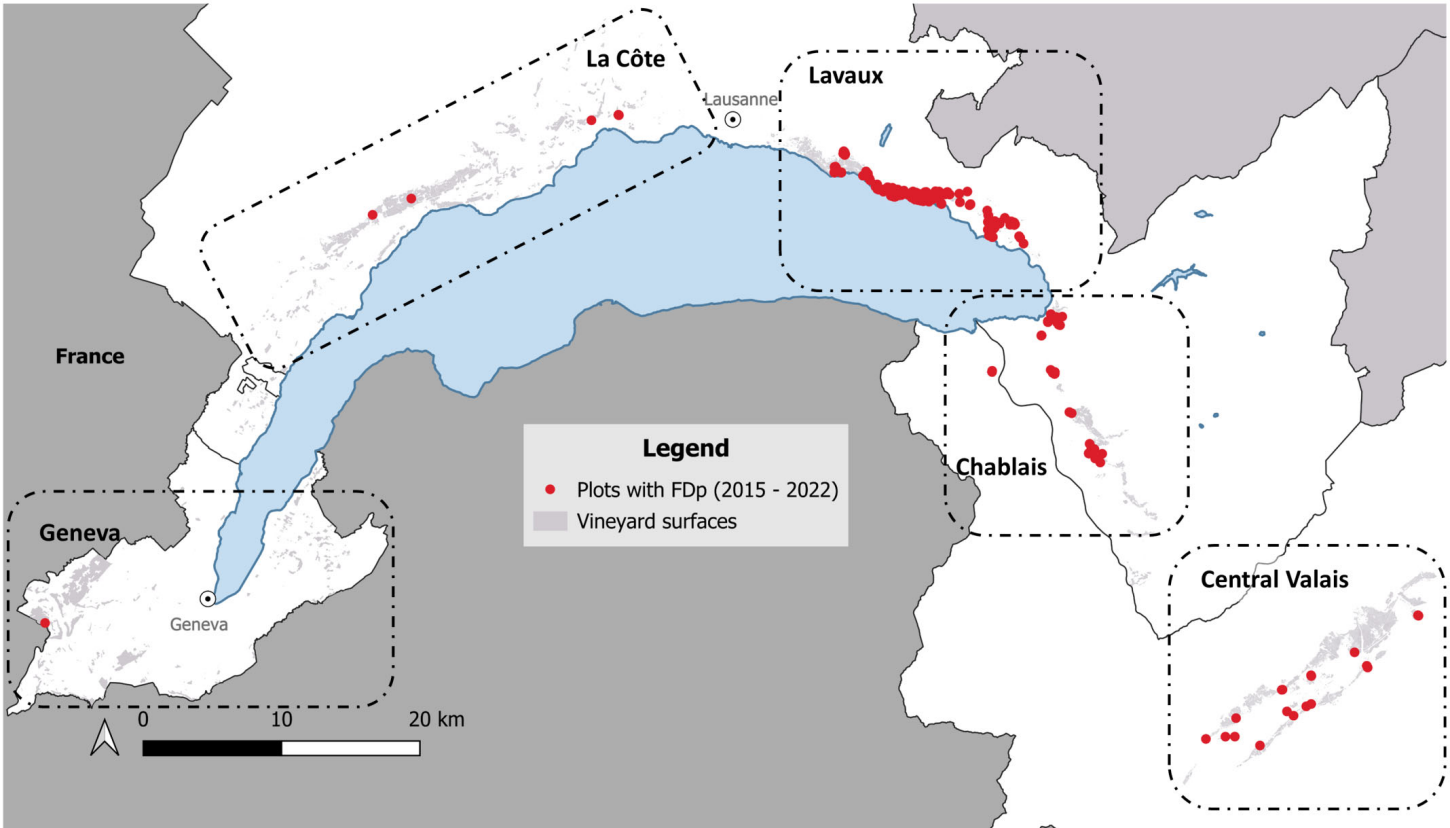
Geographical distribution of vineyards infected by Grapevine flavescence dorée phytoplasma (FDp) between 2015 and 2022 in western Switzerland. Red dots represent plots with confirmed FDp infection, and grey areas correspond to vineyard surfaces. The dashed boxes delineate the five viticultural zones considered in the study: Geneva, La Côte, Lavaux, Chablais and Central Valais.

For analytical purposes, the study area was divided into five zones: Lavaux, Chablais, Central Valais, La Côte, and Geneva (**Figure 1**).

The two earliest FD outbreaks north of the Alps were recorded in the Lavaux region in 2015 and exhibited a typical epidemic expansion in subsequent years, characterized by concentric spread. Over time, additional clusters emerged, culminating in a peak of positive cases between 2018 and 2019. Although control measures led to an important reduction in incidence, new positive cases continued to be detected thereafter.

In the Chablais region, FDp was first identified in 2020, followed by a sharp increase in cases and the emergence of multiple new outbreak sites in subsequent years. In Central Valais, an isolated case was detected in 2016, but prompt management prevented further spread, and no cases were recorded in 2017. Nevertheless, an outbreak was confirmed in 2020, with another reported the following year approximately 10 km to the south-west.

Only isolated cases have been detected to date in Geneva (2021) and La Côte (2019 and 2022), with no evidence of sustained or onward transmission in these zones. The quality of the DNA extractions allowed us to use only samples from the outbreak of 2022 in La Côte for the MLST.

### 3.3 Multilocus sequence typing

To better understand the introduction and spread of FDp in Swiss vineyards north of the Alps, a representative selection of over 700 qPCR-positive samples was analysed using a multilocus sequence typing (MLST) approach. Samples were selected based on their geographic origin and year of collection, with the aim of maximizing spatial and temporal coverage. The MLST analyses were conducted in two phases: the first phase targeted established genetic loci (*map*, *dnaK*, *vmpA* and *malG*) previously shown to exhibit sequence variability in other geographic regions. The second phase employed newly identified loci that demonstrated informative genetic variability specific to the Swiss FDp context.

#### 3.3.1 *map*, *dnaK*, *vmpA* and *malG* genotyping

Among the four loci analysed, three of them, *map*, *dnaK*, and *vmpA*, showed no genotypic variation, with all sequences displaying uniform profiles. A total of 145 samples from commercial vineyards were subjected to nested PCR and Sanger sequencing of the *map* gene. Sequence analysis revealed the exclusive presence of genotype M54. Similarly, all 77 *dnaK* sequences were identified as profile *dnak1*. The *vmpA* marker also showed no variability, with all 48 analysed samples assigned to group II.

**Figure 2:**
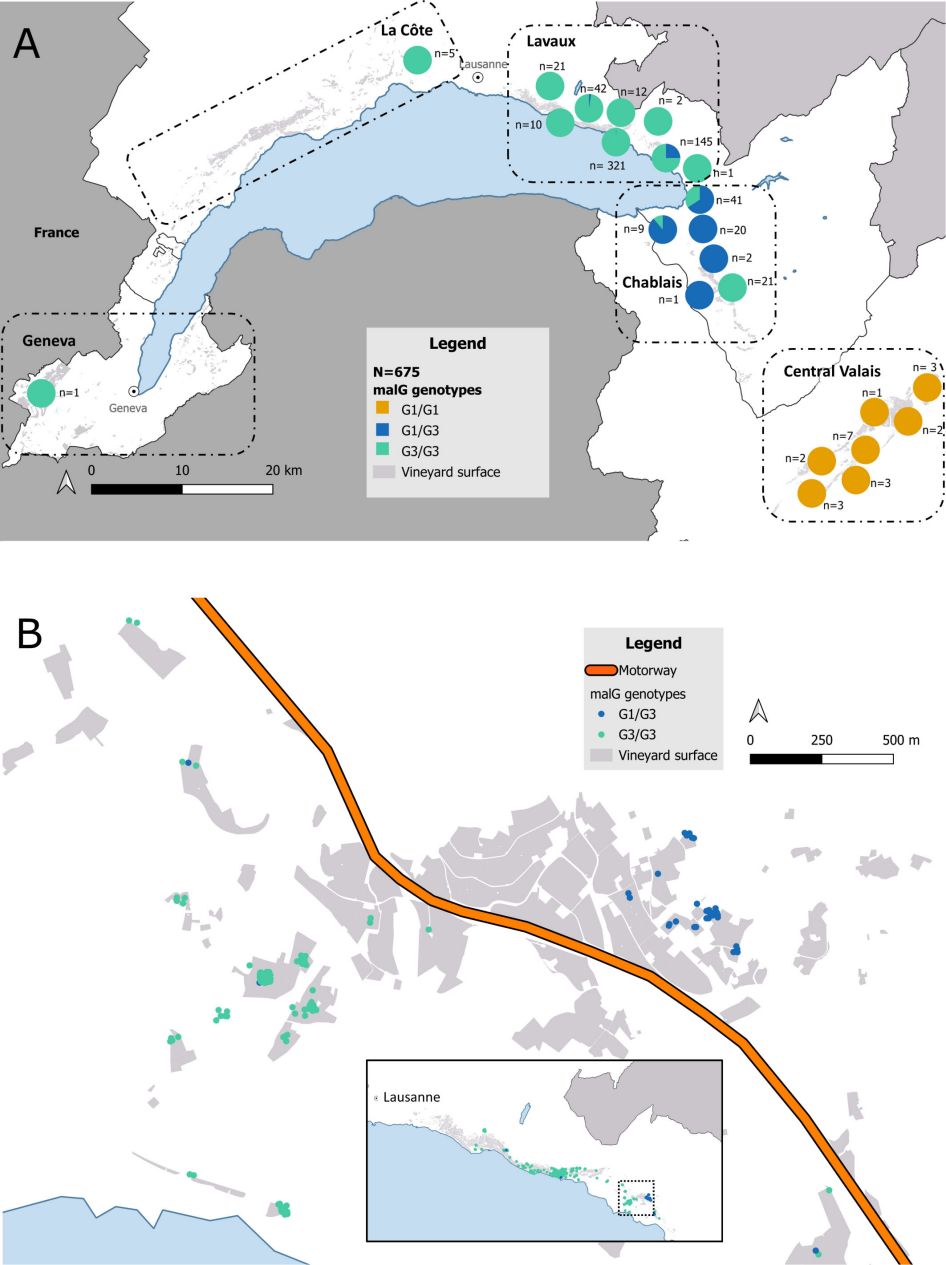
Geographic distribution of *malG* genotypes in Swiss vineyards north of the Alps. **(A)** Relative frequency of *malG* genotypes based on 675 samples collected between 2015 and 2022. Each pie chart represents samples aggregated into 3 × 3 km grid cells to improve map readability. Genotypes are classified as G1/G1 (orange), G1/G3 (blue), and G3/G3 (green), with sample counts (n) displayed next to each chart. Vineyard surfaces are shown in light grey. **(B)** Detailed view of malG genotypes in the south-eastern part of Lavaux. Points represent individual samples coloured by genotype Detailed view of malG genotypes in the south-eastern part of Lavaux. Points represent individual samples coloured by genotype (G1/G3 in blue and G3/G3 in green). The motorway is shown in orange, and vineyard surfaces are displayed in light grey.

In contrast, *malG* revealed greater genetic diversity and was therefore analysed in a larger dataset of 675 samples. Three distinct genotypes were identified: *malG* G1/G1, G3/G3 and G1/G3. Based on their geographical distribution, the infested region could be divided into three distinct zones (**Figure 2**). The *malG* G3/G3 genotype predominated in Geneva, La Côte, and almost the whole Lavaux region. The G1/G1 variant appeared to be exclusive to Central Valais. The G1/G3 genotype was most prevalent in eastern Lavaux and the Chablais area; however, the latter also exhibited the presence of several G3/G3 samples.

The resolution offered by *malG* was considered insufficient to resolve fine-scale epidemiological patterns or delineate outbreak boundaries. Therefore, we sought to increase discriminative power by incorporating additional variable SNPs located in distinct genomic regions, in order to test whether the three observed *malG* genotypes (G1/G1, G1/G3, and G3/G3) could be further differentiated.

#### 3.3.2 SNP detection and analysis

To refine genotyping resolution, novel variable genomic regions were sought within these *malG*-defined populations. For this purpose, Illumina sequencing datasets from 17 FDp-positive samples representing different geographic locations were analysed for single nucleotide polymorphisms (SNPs). Of these, six samples belonged to the *malG* G1/G3 group, nine to G3/G3, and two to G1/G1.

After read processing and mapping to the FDp-CH reference genome (Genbank CP097583), four G1/G3, six G3/G3, and both G1/G1 datasets were retained for SNP analysis based on sequence quality. Mean coverage values ranged from 6.6 ± 12.7 to 209.7 ± 46.3 for G1/G3 samples, 8.6 ± 4.2 to 119.7 ± 27.9 for G3/G3, and 34.2 ± 10.2 and 163.9 ± 7.9 for the two G1/G1 samples.

No SNPs were detected between the two G1/G1 samples, while only two were found among the six G3/G3 isolates. Of these, a synonymous SNP at the *dnaX* gene was retained, having been confirmed in two additional samples from the same plot. The second SNP, located in the *hsdR* gene, introduced a premature stop codon and was considered a likely spontaneous mutation, as it was detected in only one of the 66 samples analysed, all of which originated from the same immediate area.

In contrast, the four *malG* G1/G3 samples displayed higher variability, with 13 polymorphisms identified across the analysed datasets. Six of these were excluded from downstream analyses due to technical limitations, either unsuccessful primer design or PCR amplification failure. The remaining seven SNPs were consistently detected in multiple *malG* G1/G3 samples and retained for MLST analysis.

**Figure 3:**
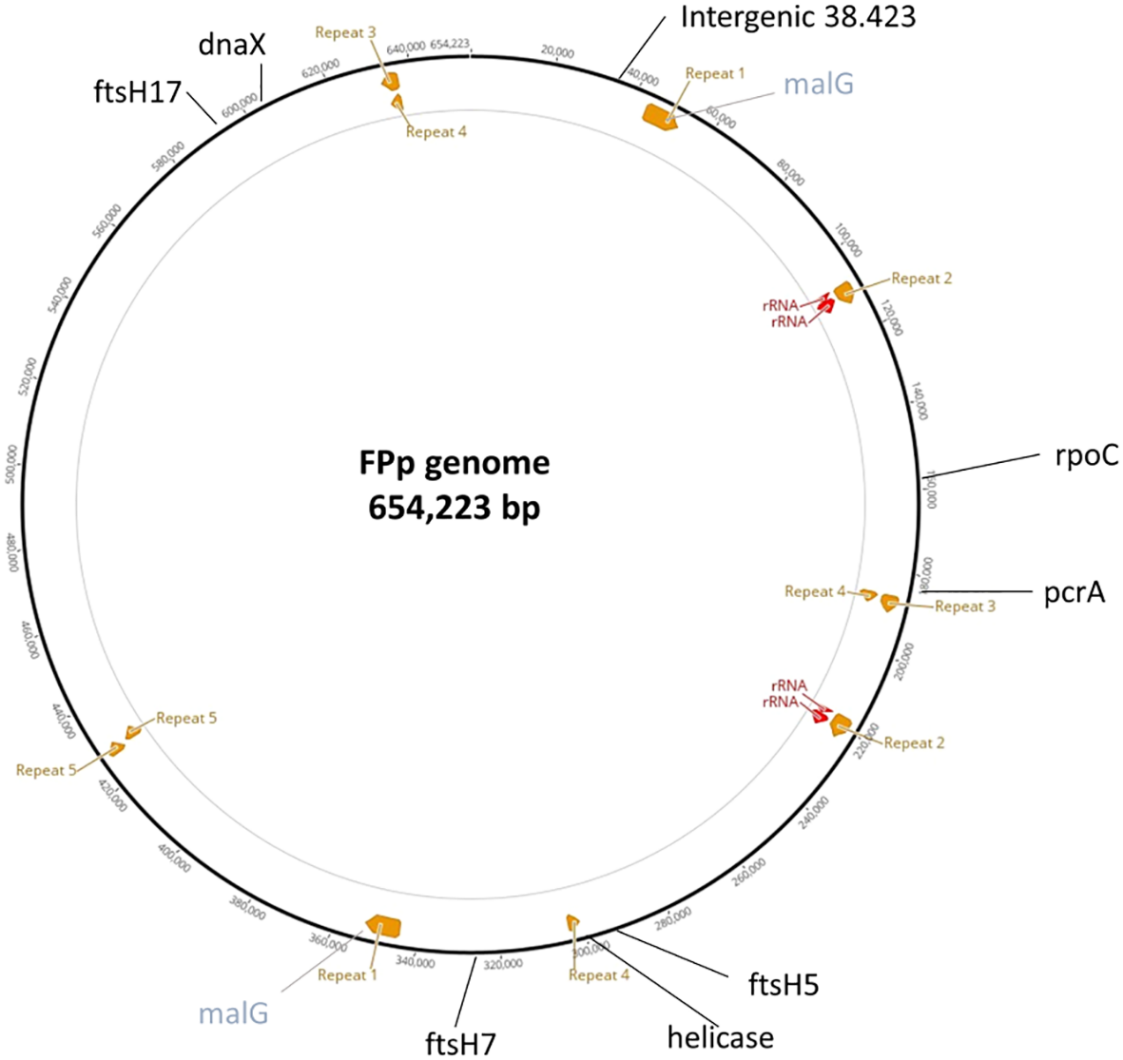
Circular representation of the Flavescence dorée phytoplasma (FDp) genome (654,223 bp), showing the positions of the eight newly identified SNP loci used for multilocus genotyping (in black) and the *malG* locus (in grey).

All selected markers were located in unique, non-repetitive regions of the genome: seven within coding sequences, five of which resulted in non-synonymous amino acid substitutions and one in an intergenic region (**Figure 3**, **Table 2**).

**Table 2:** Newly identified SNP loci in Grapevine flavescence dorée phytoplasma.

Gene name	Position on FDp strain CH (CP097583)	Nucleotide	Genotype	Coding effect
*ftsH5*	293,411	C	1	synonymous
		A	2	
*ftsH7*	323,429	C	1	non-synonymous alanine/threonine
		T	2	
*ftsH17*	593,658	C	1	non-synonymous proline/arginine
		G	2	
*dnaX*	609,876	T	1	synonymous
		C	2	
*pcrA*	184,184	C	1	non-synonymous arginine/cysteine
		T	2	
*rpoC*	159,462	G	1	non-synonymous glycine/valine
		T	2	
*helicase*	299,739	C	1	non-synonymous glycine/serine
		T	2	
Intergenic position	38,423	G	1	NA
		A	2	

For each locus, the position on the FDp-CH reference genome (GenBank CP097583) is indicated, together with the observed nucleotide variants. Each variant was assigned a numerical code (1 or 2), which was used in multilocus genotyping analyses. The predicted coding effect (synonymous or non-synonymous amino acid substitution) is also shown.

To optimise the use of resources for complete multilocus genotyping of eight loci in samples with the greatest genetic diversity, the selection of samples was first guided by the *malG* locus, which showed substantially higher variability in the G1/G3 genotype than in G3/G3 (**Table 3**). Samples were then selected based on the results of genotyping individual SNP loci across a broad set of samples representing different years and plots. The following numbers of samples were analysed per SNP locus: n = 119 (*ftsH5*), n = 86 (*ftsH7)*, n = 114 (*ftsH17*), n = 86 (*pcrA*), n = 89 (*dnaX*), n = 89 (*rpoC*), n = 77 (*helicase*), and n = 85 (the intergenic position at 38,423). Based on this preliminary screening, full genotyping was performed at all loci for 65 samples with maximised genetic diversity. Sampling effort was increased in plots and regions showing higher variability during the initial screening, while fewer samples were selected from regions with more homogeneous profiles. For example, the *malG* G3/G3 genotype was largely predominant in Lavaux (525 out of 564 analysed samples), showing limited SNP variability in both Illumina and MLST analyses. In contrast, the *malG* G1/G3 genotype was predominant in Chablais (78 out of 94 analysed samples), displaying higher SNP variability. Consequently, this guided the selection of a larger number of samples for complete multilocus genotyping in Chablais than in Lavaux. In Geneva, La Côte and Central Valais, the number of samples showing symptoms and genetic diversity was limited at the time of the study, resulting in 11 fully sequenced samples.

**Table 3:** SNP-based multilocus genotypes of Grapevine flavescence dorée phytoplasma (FDp) within the three profiles defined by the *malG* locus.

Multilocus genotype	*malG*	*ftsH5*	*ftsH7*	*ftsH17*	*dnax*	*pcrA*	*rpoC*	*helicase*	Intergenic 38,423	# samples	% within the *malG* group
CH_A	G3/G3	1	1	1	1	1	1	1	1	16	88.89%
CH_B	G3/G3	1	1	1	1	2	2	1	1	2	11.11%
CH_C	G3/G3	1	1	1	2	1	1	1	1	2	11.11%
CH_D	G1/G3	1	1	1	1	2	2	1	1	21	56.76%
CH_E	G1/G3	1	1	1	1	1	1	1	1	3	8.11%
CH_F	G1/G3	1	2	1	1	1	1	2	2	7	18.92%
CH_G	G1/G3	1	2	2	1	1	1	2	2	2	5.41%
CH_H	G1/G3	2	2	2	1	1	1	2	2	4	10.81%
CH_I	G1/G1	1	1	1	1	1	1	1	1	8	100%

For each SNP locus, the numeric codes correspond to the genotype designations given in **Table 2**. Combinations of these genotypes define multilocus profiles. The number of samples (n) and their relative frequency within their *malG* group (%) are indicated for each profile.

### 3.4 Genotypes identification and geographic distribution

**Figure 4:**
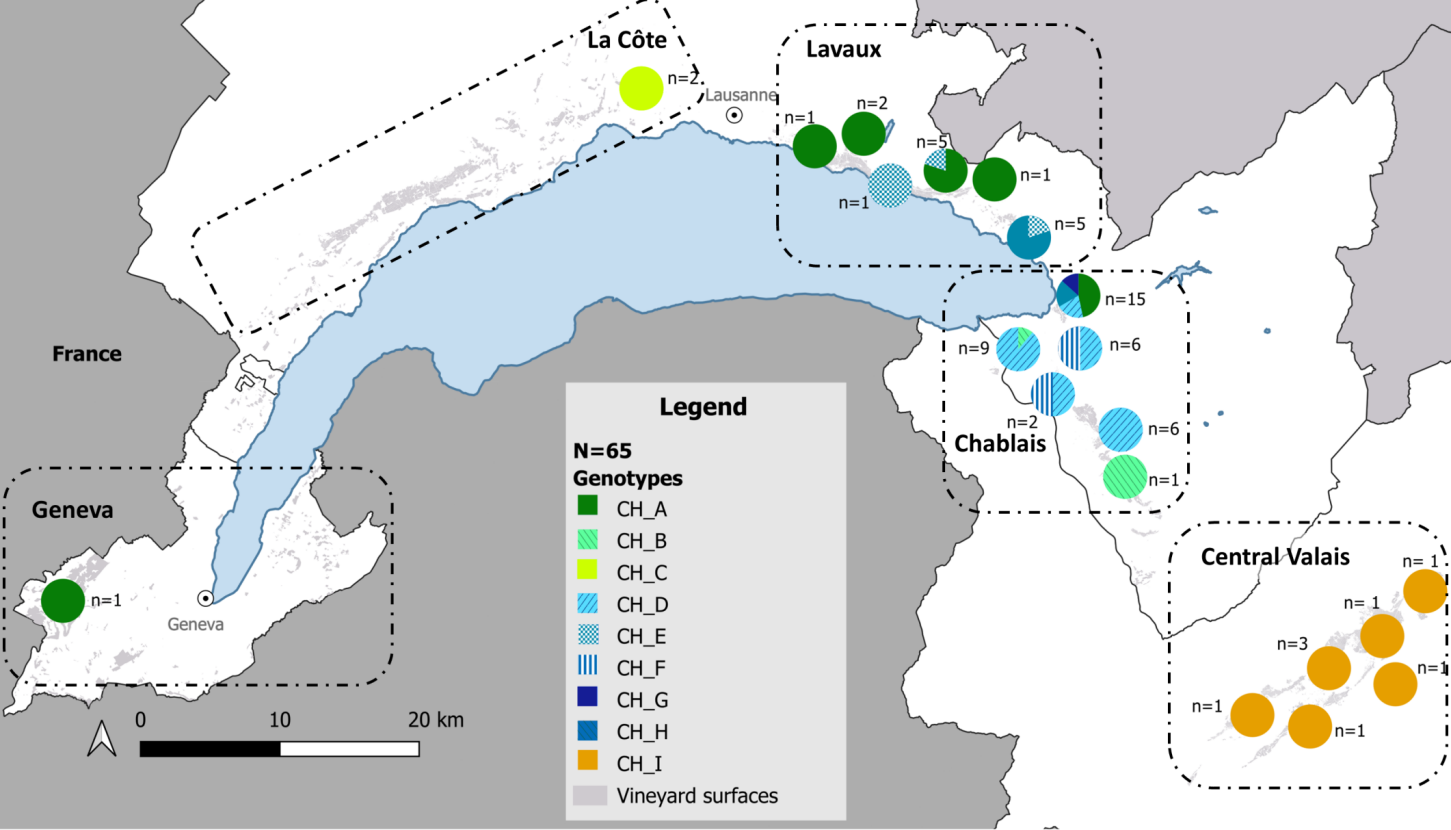
Geographic distribution of multilocus genotypes of Grapevine flavescence dorée phytoplasma (FDp) in Swiss vineyards north of the Alps. Pie charts show the relative frequency of SNP-based multilocus genotypes at each location, based on 65 representative samples collected between 2015 and 2022. Each pie chart represents samples grouped in 3×3km grid cells to improve map readability. Genotypes (CH_A to CH_I) are indicated by different colours, as shown in the legend. Sample counts (n) are indicated next to each chart. Vineyard surfaces are shown in light grey.

A total of nine distinct genotypes were identified based on all observed combinations of variable loci present within individual samples (**Table 3**). Genotypic differentiation was achieved using the previously described *malG* locus (Rossi et al., 2019), in combination with the eight newly detected SNPs. Two genotypes were predominant: CH_A (corresponding to the *malG* G3/G3 matching the published FDp whole genome sequence; (Debonneville et al., 2022)) and CH_D. This was followed by CH_I, the sole genotype detected in Central Valais region, where all eight samples from different locations exhibited this genotype (**Figure 4**). Genotype CH_F was primarily found in the eastern area of Lavaux, whereas the remaining genotypes appeared to be rare.

Regional genotypic analyses showed contrasting levels of genetic diversity and spatial structure among FDp populations across Swiss vineyards north of the Alps (**Figure 4**). Chablais showed the highest diversity, with six multilocus genotypes detected. In contrast, only three genotypes (CH_A, CH_E, and CH_F) were found in Lavaux. SNP analysis within the *malG* G3/G3 group revealed that the FDp population in La Côte differed from that in Lavaux by one base. In Geneva, the quality of the Illumina sequencing for the *malG* G3/G3 sample was insufficient to allow SNP-based genotyping, but the absence of the SNP identified in the 2020 outbreak of La Côte was confirmed. Finally, the population structure in Central Valais remained unchanged, as no SNPs were detected within the *malG* G1/G1 group. Interestingly, CH_E was detected in only three samples within a predominantly CH_A area in Lavaux. Both genotypes share the same combination of newly detected SNPs and differ solely at the *malG* locus. A similar pattern was observed with genotype CH_B, found in two samples within a CH_D-dominated region in Chablais, also sharing SNP combinations and differing only by the *malG* locus.

## 4 Discussion

The primary objective of this study was to elucidate the origin and spread of FDp in Swiss vineyards north of the Alps. To this end, we analysed FDp whole-genome variability to develop new variable markers, which were integrated with known loci into a MLST analysis. Over 700 FDp-positive samples were screened for selected loci, while a complete MLST analysis was conducted on 65 representative samples.

### 4.1 Introductory events

The *map* locus proved highly informative for investigating the origin of FDp in the studied region, where a single *map* genotype, M54, was consistently detected across all samples spanning more than 100 km. This genotype is almost exclusively associated with the *Vitis vinifera*–*Scaphoideus titanus* pathosystem (Malembic-Maher et al., 2020) and has rarely been reported in alternative perennial hosts (Casati et al., 2017; Moussa et al., 2023; Rigamonti et al., 2023). Moreover, the initial outbreaks of FD occurred in the middle of continuous vineyard areas, far from potential alternative hosts and geographically isolated from the closest infested zones. Taking into account all this information, we hypothesize that FDp was most probably introduced primarily through infected grapevine planting material. Nonetheless, further investigations are ongoing to clarify the potential role of alternative insect vectors and plant hosts in local FDp persistence and spread.

This low diversity in the *map* locus contrasts sharply with observations in other European regions, where much higher diversity has been documented over relatively short distances. For instance, four *map* genotypes were identified within 240 km in Croatian grapevines (Plavec et al., 2019), six within 50 km in Slovenia (Kogej Zwitter et al., 2023), and four within 140 km in France’s Aquitaine region (Malembic-Maher et al., 2020). Elevated genotype richness, particularly in the Balkans, likely reflects the presence of FDp in alternative plant hosts, which have been hypothesized to be the origin of the epidemics in Serbia and Montenegro (Krstić et al., 2022 and Radonjić et al., 2023, respectively). In the studied region, this low diversity might be due to scarce introductory events together with the recent introduction of the phytoplasma.

### 4.2 MLST, genomic variability and marker development

MLST analysis using the additional known loci *vmpA*, *dnaK and malG* confirmed very low genotypic diversity in the FDp populations studied: no variation was detected in *vmpA*, or *dnaK*, and only limited variation was observed at the *malG* locus, identifying three genotypes (G1/G1, G1/G3, and G3/G3). This contrasts with findings from other regions: for instance, Rossi et al. (2019) reported up to 11 *malG* types in grapevines in the Piedmont area.

To better resolve local epidemic dynamics and assess potential links between geographically close outbreaks, we sought to increase the genotyping resolution beyond what was possible with *malG* alone. Indeed, the three *malG* genotypes (G1/G1, G1/G3, and G3/G3) suggested only a limited number of populations, which did not fully account for the observed spatial complexity and temporal progression of the epidemic. We therefore explored genome-wide SNP variation within these groups; an approach that, to our knowledge, is novel in FDp population studies. This enabled the identification of eight additional variable loci, primarily within the G1/G3 group, increasing resolution from three to nine multilocus genotypes.

### 4.3 Spread dynamics and epidemiological patterns

Using the refined genotyping approach, only one genotype was detected from geographically distant samples in Central Valais, consistent with a single introduction followed by local spread, likely mediated by infected *S. titanus*. The initially scattered distribution observed until 2022 may be attributable to delayed symptom expression caused by the latency period of phytoplasma development within the host or by incomplete surveillance. By 2023, vineyards previously free of infection between positive plots had become affected (data not shown).

In Lavaux, the two dominant genotypes showed clear geographic segregation, likely influenced by a motorway acting as a physical barrier to insect movement (**Figure 2b**). CH_E was detected in only three samples from spatially scattered locations within an area otherwise dominated by CH_A. Notably, these CH_E samples carried a *malG* G1/G3 genotype, contrasting with the surrounding population, which was overwhelmingly characterised by *malG* G3/G3. Interestingly, CH_E shares the same SNP profile as both CH_A (from Lavaux) and CH_I (from Valais), differing only at the *malG* locus. This close genetic similarity suggests a recent divergence or a shared ancestral origin.

The rarity and scattered presence of CH_E on three different locations in Lavaux region raise questions about its origin. Given its extremely low frequency and its occurrence within a largely homogeneous area, the most parsimonious explanation is the introduction of this genotype through planting material, either via a shared source used for vine replacement or through a single introduction event followed by local dissemination. Its low frequency might reflect limited dispersal or establishment capacity. Alternative explanations cannot be excluded, and CH_E could have emerged locally from a CH_A background through changes affecting one of the two *malG* copies, converting from *malG*3 to *malG*1, potentially within the insect vector. Rossi et al. (2019) reported that approximately 50% of the 183 *malG* types detected in their study were found exclusively in insects, and suggested a high variation rate of FDp populations within the vector as one possible explanation. Nevertheless, the plausibility of a direct *malG*3-to-*malG*1 shift is highly questionable, as it would require at least four nucleotide changes. Homologous recombination occurring in the insect hosting different variants of *malG* could also theoretically be envisaged, but phytoplasmas generally show poor recombination capacity (Bai et al., 2006), and the FDp genome lacks potential mobile units (PMUs) typically involved in such processes (Debonneville et al., 2022). In the absence of experimental evidence, the origin of these strains, which differ exclusively at the *malG* locus, remains unresolved. Intriguingly, a similar situation was observed in the Chablais region, where the rare genotype CH_B coexisted with the predominant CH_D, sharing identical SNP profiles and differing only at the *malG* locus.

The Chablais region showed a more complex, mixed distribution of six genotypes across disconnected vineyard patches separated by distances exceeding 3 km, which surpasses the currently known active flight range of *S. titanus*. Studies have demonstrated that *S. titanus* typically flies within 30 m for 80% of its movements, with occasional flights up to 330 m (Lessio et al., 2014), and recent evidence supports active and common movement up to at least 210 m (Petit, 2023). Moreover, human-assisted movement via agricultural machinery has been shown to transport *S. titanus* over distances up to 2.5 km (Chavarri-Padilla, 2023), and wind can also act as a passive dispersal mechanism (Petit, 2023). Vine growers may manage several spatially dispersed plots across different villages, use shared agricultural machinery (including service providers operating the same equipment across multiple vineyards), and exchange planting material for dead vine replacements. However, detailed plot-level information on these practices is generally not recorded and was not available for the vineyards where positive samples analysed in this study were identified. The causes of the complex epidemiological dynamics observed in the Chablais region remain uncertain. Nevertheless, the hypothesis of a more pronounced influence of region-specific passive diffusion processes points to the possible role of local socio-agricultural determinants.

Overall, these spatial and genetic patterns are consistent with a scenario in which the spread of FDp in Swiss vineyards may be shaped by two main forces: first, the natural epidemiological expansion of the phytoplasma via its insect vector in regions where grapevine cultivation is spatially continuous, such as Lavaux, and second, a combination of anthropogenic and ecological drivers of dispersal. Anthropogenic factors may include the movement of infected planting material, vineyard management practices, and physical barriers such as roads or motorways, while ecological drivers include wind-mediated vector dispersal and the fragmented structure of certain viticultural landscapes, as observed in Chablais. In this context, the presence of rare genotypes in spatially distant or disconnected plots suggests that vector movement alone may not fully account for the observed patterns. Rather, the interplay between local environmental conditions and human-mediated practices could have contributed to shaping the epidemic landscape. Together, these observations highlight the value of combining molecular surveillance with landscape-level epidemiological monitoring to improve our understanding of FDp spread and inform disease management strategies.

## 5 Conclusion

This MLST analysis brings evidence that FDp in Swiss vineyards north of the Alps was most likely introduced by infected planting material. It also highlights the combination of natural and anthropogenic forces in the dissemination of the disease. Therefore, our results underline the importance of planting healthy propagating material and strategically managing viticultural practices to prevent the spread of FD. Finally, this work proposes new SNPs for fine-scale characterisation of homogeneous FDp populations, which can help to unravel local dynamics of its spread. Having been applied to the Swiss FDp population north of the Alps, these new SNP markers could now be tested in other viticultural regions to evaluate their broader relevance and to gain further insight into FDp dissemination at larger geographic scales.

## Data Availability

Reference sequences generated by Sanger sequencing from PCR products amplified with the published primer sets were deposited in the NCBI database. For each locus, one reference sequence was deposited for each variant (variant 1 and variant 2), with the following accession numbers: Locus_variant Accession number, 38423_1 (PX849515), 38423_2 (PX849516), dnaX_1 (PX849517), dnaX_2 (PX849518), ftsH5_1 (PX849519), ftsH5_2 (PX849520), ftsH7_1 (PX849521), ftsH7_2 (PX849522), ftsH17_1 (PX849523), ftsH17_2 (PX849524), helicase_1 (PX849525), helicase_2 (PX849526), pcrA_1 (PX849527), pcrA_2 (PX849528), rpoC_1 (PX849529), rpoC_2 (PX849530). Illumina sequencing data supporting the conclusions of this study has been deposited in the NCBI Sequence Read Archive (SRA) repository, PRJNA1417603.

## Footnotes

1 https://web.expasy.org/translate/.
